# SimSon: simple contrastive learning of SMILES for molecular property prediction

**DOI:** 10.1093/bioinformatics/btaf275

**Published:** 2025-05-08

**Authors:** Chae Eun Lee, Jin Sob Kim, Jin Hong Min, Sung Won Han

**Affiliations:** Department of Industrial and Management Engineering, Korea University, Seoul 02841, Republic of Korea; Department of Industrial and Management Engineering, Korea University, Seoul 02841, Republic of Korea; Department of Industrial and Management Engineering, Korea University, Seoul 02841, Republic of Korea; Department of Industrial and Management Engineering, Korea University, Seoul 02841, Republic of Korea

## Abstract

**Motivation:**

Molecular property prediction with deep learning has accelerated drug discovery and retrosynthesis. However, the shortage of labeled molecular data and the challenge of generalizing across the vast chemical spaces pose significant hurdles for leveraging deep learning in molecular property prediction. This study proposes a self-supervised framework designed to acquire a Simplified Molecular Input Line Entry System (SMILES) representation, which we have dubbed Simple SMILES contrastive learning (SimSon). SimSon was pre-trained using unlabeled SMILES data through contrastive learning to grasp the SMILES representations.

**Results:**

Our findings demonstrate that contrastive learning with randomized SMILES enriches the ability of the model to generalize and its robustness as it captures the global semantic context at the molecular level. In downstream tasks, SimSon performs competitively when compared to graph-based methods and even outperforms them on certain benchmark datasets. These results indicate that SimSon effectively captures structural information from SMILES, exhibiting remarkable generalization and robustness. The potential applications of SimSon extend to bioinformatics and cheminformatics, encompassing areas such as drug discovery and drug–drug interaction prediction.

**Availability and implementation:**

The source code is available at https://github.com/lee00206/SimSon.

## 1 Introduction

Recent advances in machine learning have sparked the interest in computational approaches for cheminformatics and bioinformatics, particularly in drug discovery, where development costs can reach 2.8 billion collars (Wieder *et al.* 2020). Moreover, the success rate for drug design stands at ∼6.2% (Vamathevan *et al.* 2019), with development taking anywhere from 5.9 to 13.1 years (Kolluri *et al.* 2022). These challenges make machine learning an appealing tool in the pharmaceutical sector, given its automated nature and predictive capabilities, which can enhance the efficiency of drug design. Machine learning has made significant strides in drug discovery (Chen *et al.* 2018, Gómez-Bombarelli *et al.* 2018, Lim *et al.* 2018), binding affinity prediction (Öztürk *et al.* 2018, Stepniewska-Dziubinska *et al.* 2018), molecular property prediction (Klambauer *et al.* 2017, Schütt *et al.* 2018, Lu *et al.* 2019), and various other domains.

Molecular property prediction is essential in various fields, including drug discovery, retrosynthesis, biophysics, and quantum mechanics (Zhang *et al.* 2021, Li *et al.* 2022). Machine learning accelerates this process by enabling rapid and cost-effective predictions, reducing the need for expensive in vivo experiments. However, supervised learning methods require large, high-quality labeled datasets to achieve high accuracy, which is particularly challenging in cheminformatics and bioinformatics, where data acquisition depends on costly and time-consuming wet-lab experiments (Rong *et al.* 2020, Zhang *et al.* 2021, Fang *et al.* 2022).

To address data scarcity, self-supervised learning has emerged as a promising alternative by leveraging large-scale unlabeled molecular data. Inspired by its success in computer vision (CV) and natural language processing (NLP) (Larsson *et al.* 2017, Radford *et al.* 2018, Fang *et al.* 2020), recent studies have applied self-supervised learning to molecular property prediction (Chithrananda *et al.* 2020, Rong *et al.* 2020, Zhang *et al.* 2021, Fang *et al.* 2022). However, many existing approaches primarily capture local atomic and bond-level semantics, often overlooking global molecular semantics, which are crucial for accurately modeling interactions between functional groups and determining molecular properties.

Contrastive learning has emerged as a powerful self-supervised approach for molecular representation learning (Chen *et al.* 2020, Fang *et al.* 2020, Wang *et al.* 2022a), aiming to improve molecular data representations and capture molecular-level semantics. By learning to pull similar molecules closer while pushing dissimilar ones apart (Hadsell *et al.* 2006), contrastive learning enhances model robustness. As a result, recent studies have incorporated this technique into molecular property prediction (Guo *et al.* 2021, Zhu *et al.* 2021, Wang *et al.* 2022b). However, despite its effectiveness in capturing molecular semantics, contrastive learning still faces challenges in generalizing across the vast chemical space, which encompasses ∼10^60^ potential molecules, while only 10^8^ have been experimentally identified (Wu *et al.* 2019). This immense gap highlights the difficulty of learning representations that extend beyond known compounds, underscoring the need for improved generalization techniques.

Molecular representation methods are crucial for improving generalization, with common formats including fingerprints, Simplified Molecular Input Line Entry System (SMILES) (Weininger 1988), the IUPAC International Chemical Identifier (InChI) (Heller *et al.* 2015), and molecular graphs. While graph-based representations encode structural information (Rong *et al.* 2020, Zhang *et al.* 2021, Fang *et al.* 2022), they rely on predefined molecular features, limiting flexibility. In contrast, SMILES is widely used in machine learning due to its compactness and efficiency (O’Boyle 2012, David *et al.* 2020), making it the default format in chemical databases (Irwin and Shoichet 2005, Mendez *et al.* 2019, Kim *et al.* 2021).

Despite its advantages, SMILES has limitations, including loss of topological information and non-unique representations, which introduce ambiguity (David *et al.* 2020, Fang *et al.* 2022). Sequence-based models like Recurrent Neural Networks (RNNs) and Long Short-Term Memory (LSTMs) (Hochreiter and Schmidhuber 1997, Sherstinsky 2020) struggle to capture complex molecular structures, while Transformer-based models leverage self-attention to learn long-range dependencies, improving molecular property prediction (Zheng *et al.* 2019, Chithrananda *et al.* 2020). Additionally, randomized SMILES (Bjerrum 2017) enhances generalization by generating multiple equivalent representations of the same molecule, mitigating biases from specific SMILES generation rules (Arús-Pous *et al.* 2019).

Inspired by the success of contrastive learning and self-attention mechanisms in capturing SMILES structural information, we propose **Sim**ple **S**MILES c**on**trastive learning (SimSon), a self-supervised framework for learning SMILES representations. SimSon identifies similarities between different SMILES of the same molecule and dissimilarities between different molecules, using randomized SMILES generated through enumeration-based augmentation. This approach improves generalization and robustness by capturing global molecular semantics. We pre-trained the model on one million unlabeled molecules and fine-tuned it on downstream molecular property prediction tasks. The model’s robustness was assessed using cosine similarity between embeddings of different SMILES representations of the same molecule. The contributions of this study can be summarized as follows.

- We propose SimSon, a self-supervised framework that learns SMILES representations via contrastive learning using only SMILES data. SimSon can be trained on a diverse set of SMILES, eliminating the need for specific SMILES for molecular representation conversion.
- We illustrate that pre-training SimSon with randomized SMILES improves the generalization capability of the model, with SimSon achieving top performance in four out of seven benchmark datasets.
- We demonstrate that contrastive learning with randomized SMILES enables the capture of enhanced global semantics at the molecular level. Strong cosine similarities between the embedding vectors of different SMILES representations of the same molecule showcase the robustness of SimSon.

## 2 Preliminaries and related work

### 2.1 SMILES molecular representation

**Figure 1.**
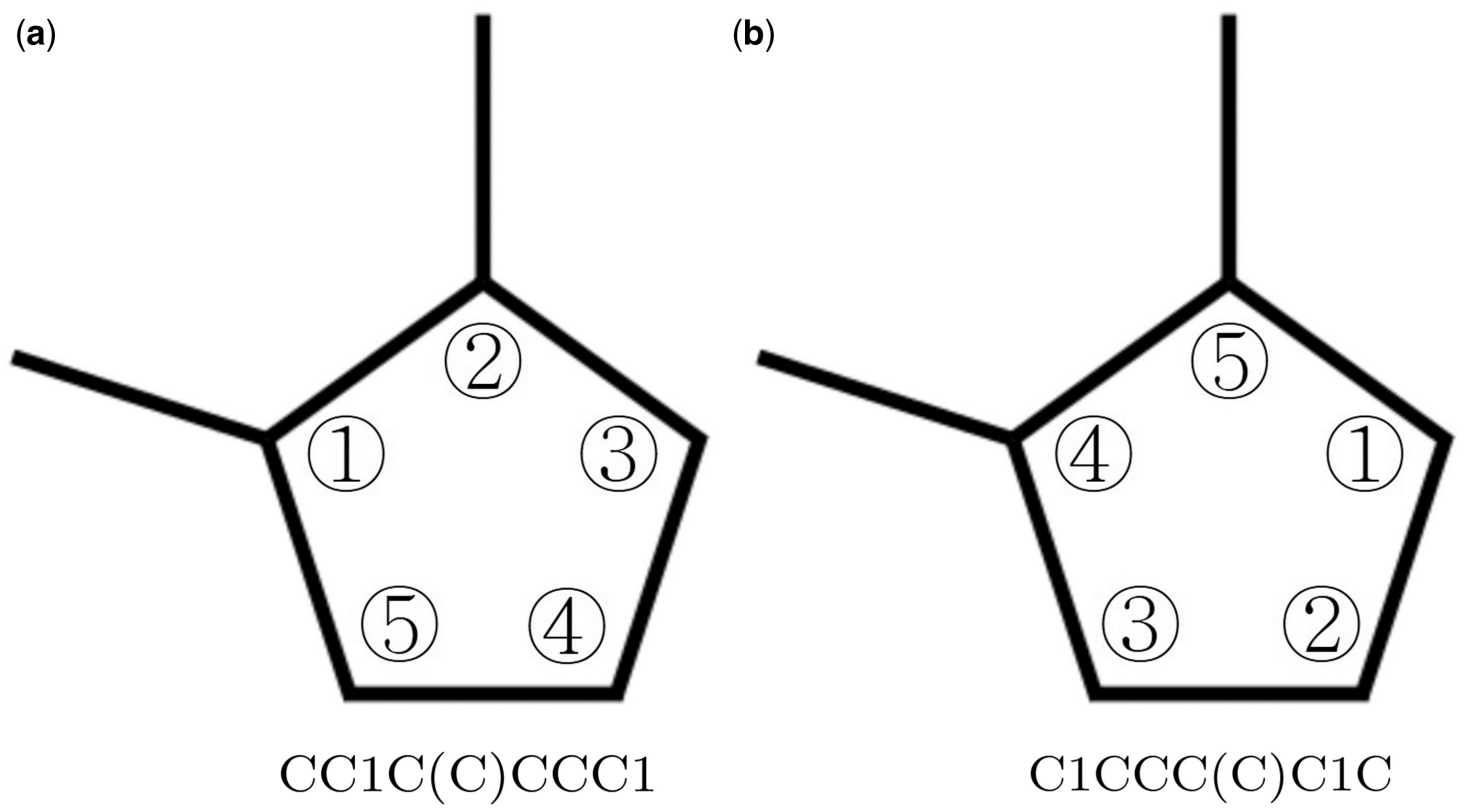
Dimethylcyclopentane with different SMILES depending on atom numbering. (a) SMILES with methyl groups connected to one and two carbons. (b) SMILES with methyl groups connected to four and five carbons.

SMILES is a widely used string-based representation of molecular structures, where atoms and bonds are encoded as characters in a linear sequence. However, SMILES exhibits inherent ambiguity, as a single molecule can have multiple valid representations depending on atom numbering and generation algorithms (Fig. 1). This variability poses challenges for sequence-based models such as RNN and LSTM, which process SMILES as sequential data and may struggle to capture structural relationships accurately (Fang *et al.* 2022).

To address these challenges, prior works (Zheng *et al.* 2019, Chithrananda *et al.* 2020) have explored self-attention mechanisms to better capture the syntax and semantics of SMILES. Additionally, a data augmentation technique known as randomized SMILES (Bjerrum 2017) has been proposed, which generates multiple equivalent SMILES representations for a molecule by randomizing atom order. This technique improves generalization and prevents bias from specific generation algorithms, allowing models to learn a more robust molecular representation (Arús-Pous *et al.* 2019).

### 2.2 Graph molecular representation

Graph representations model molecules as graphs, where atoms are nodes and bonds are edges, often enriched with structural features such as chirality, hybridization, and formal charge. Despite being inherently 2D structures, graphs can incorporate 3D information, making them widely used in molecular property prediction tasks (David *et al.* 2020). However, constructing graphs can be challenging, as the molecular features included depend on the graph generation algorithm, and some features may be unavailable from the raw data. Another drawback of graph representations is their high memory consumption, as storing and processing graph structures require significantly more computational resources compared to sequence-based representations. Additionally, most graph-based representations are derived from SMILES, meaning molecular structures are first encoded as SMILES before being converted into graphs.

Given these factors, leveraging SMILES as a molecular representation remains a viable approach, especially when advanced techniques are used to extract molecular semantics effectively. This study focuses solely on SMILES-based learning, demonstrating that a well-designed model can capture molecular structure and properties efficiently.

### 2.3 Predictive self-supervised learning

Self-supervised learning is a technique used to train models for learning data representations without relying on labeled data. As a result, many studies have used self-supervised learning when dealing with a limited amount of available data. One common method within self-supervised learning is the predictive approach, where models learn data representations by making predictions on masked tokens, atoms, or bonds. For instance, ChemBERTa (Chithrananda *et al.* 2020) predicts masked tokens to acquire SMILES representations, GROVER (Rong *et al.* 2020) focuses on predicting masked atom or bond attributes to learn molecular graphs. MGSSL (Zhang *et al.* 2021) takes a step further by predicting masked atoms or bonds and generating motifs, effectively leveraging both molecular graphs and motifs. HiMol (Zang *et al.* 2023) extends this approach to make predictions at node, motif, and graph levels, incorporating hierarchical multi-order information of molecules.

### 2.4 Contrastive self-supervised learning

Contrastive learning represents another approach to self-supervised learning. It operates by learning data representations through the maximization of similarities between two distinct vector representations of the same object, using a contrastive loss function (Chen *et al.* 2020).

Early works explored contrastive learning for molecular representation by integrating multiple molecular notations. DMP (Zhu *et al.* 2021) applied contrastive learning to jointly learn both graph-based and SMILES representations, while MMDeacon (Guo *et al.* 2021) extended this approach by learning contrastive representations between SMILES and IUPAC notations.

Later, MolCLR (Wang *et al.* 2022b) introduced contrastive learning with graph-based augmentations, enhancing molecular graphs by randomly masking atoms or deleting bonds before computing contrastive loss. GraphMVP (Liu *et al.* 2021) expanded upon this by performing contrastive learning between 2D and 3D molecular representations, maximizing mutual information between them to improve molecular understanding. KANO (Fang *et al.* 2023) further refined contrastive learning-based pre-training by introducing element-guided augmentation and leveraging functional prompts derived from functional group knowledge. MolMVC (Huang *et al.* 2024) then extended multi-view contrastive learning by integrating 1D (SMILES), 2D (graph-based), and 3D (spatial) molecular structures, capturing comprehensive molecular representations. Most recently, MolLM (Tang *et al.* 2024) built upon these advancements by optimizing contrastive learning between 2D and 3D molecular structures, further improving the alignment between different molecular views.

Many previous studies (Rong *et al.* 2020, Liu *et al.* 2021, Zhang *et al.* 2021, Wang *et al.* 2022b, Fang *et al.* 2023, Zang *et al.* 2023) have utilized a graph-based molecular representation, while others, including (Chithrananda *et al.* 2020, Guo *et al.* 2021, Zhu *et al.* 2021, Huang *et al.* 2024, Tang *et al.* 2024), have directly processed SMILES representations. In contrast to these existing methodologies, SimSon focuses solely on training with SMILES data. SimSon is specifically designed to enhance the learning of SMILES representations without the need for conversion into alternative molecular representations. It accomplishes this by using contrastive learning with randomized SMILES, which allows it to capture global semantics at the molecular level and improve generalization within the molecular space, as demonstrated in (Arús-Pous *et al.* 2019). SimSon is a straightforward yet effective approach for learning SMILES representations, achieved through the combination of the SMILES enumeration technique and contrastive learning with extensive unlabeled data.

## 3 Methodology

### 3.1 Framework

**Figure 2.**
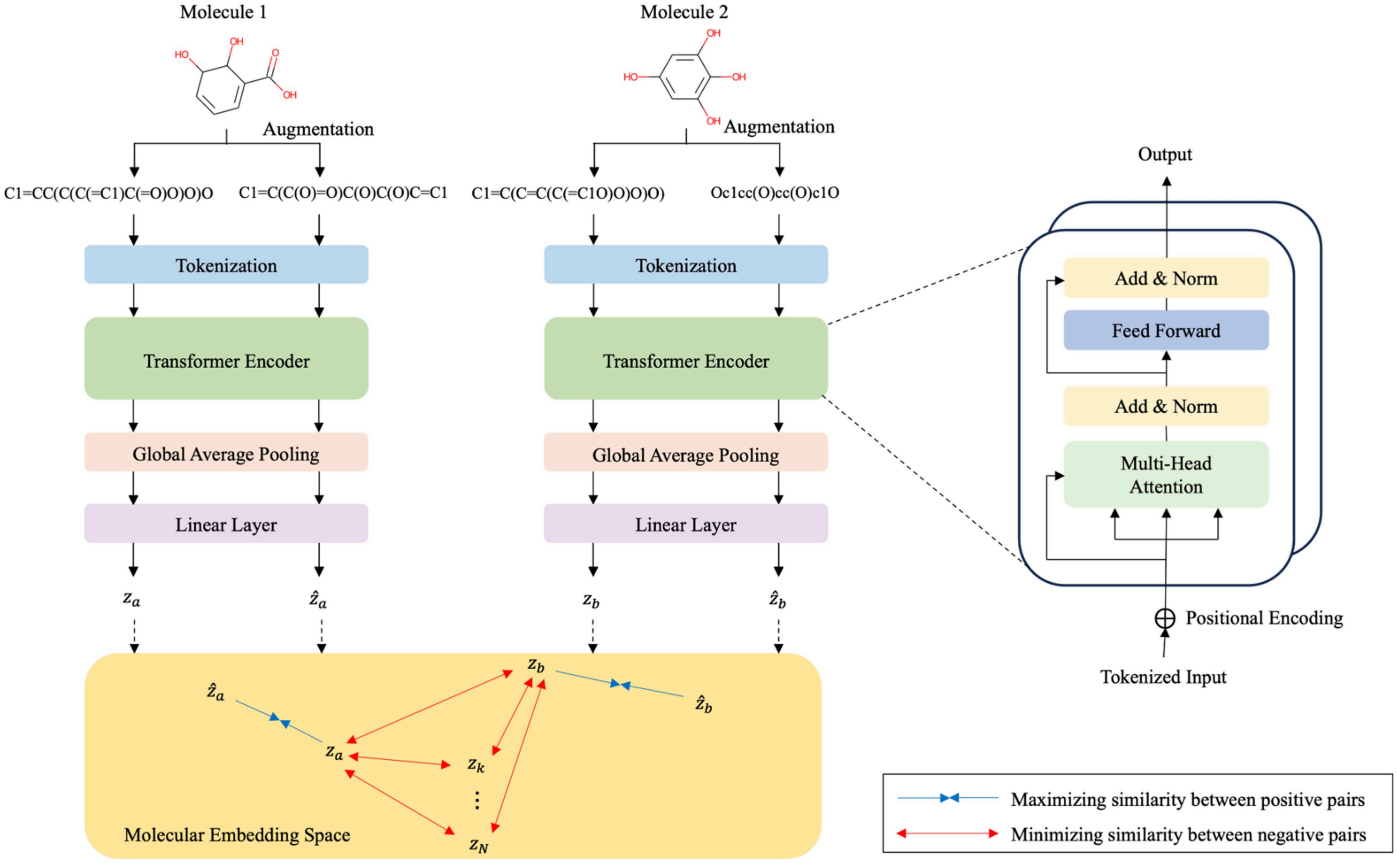
Overview of SimSon. SMILES of Molecule 1 and Molecule 2 are augmented by randomizing atom numbers. Augmented SMILES are tokenized, and positional encoding is added prior to feeding it into the Transformer encoder. The model outputs z${}_{a}$ and ${\hat{z}}_{a}$ from Molecule 1 and z${}_{b}$ and ${\hat{z}}_{b}$ from Molecule 2. The outputs of the model are used to compute the contrastive loss. The contrastive loss function maximizes the similarities between the positive pairs and minimizes the similarities between the negative pairs. The positive pairs are the SMILES indicating the same molecule. The negative pairs are the SMILES of different molecules in a mini-batch. That is, (z${}_{a}$, ${\hat{z}}_{a}$) and (z${}_{b}$, ${\hat{z}}_{b}$) are the positive pairs whereas (z${}_{a}$, z${}_{b}$) represents the negative pair.

In this section, we describe the SimSon framework, including data processing, model architecture, and pre-training. The overall framework of SimSon is illustrated in Fig. 2.

### 3.2 Data pre-processing

**Figure 3.**
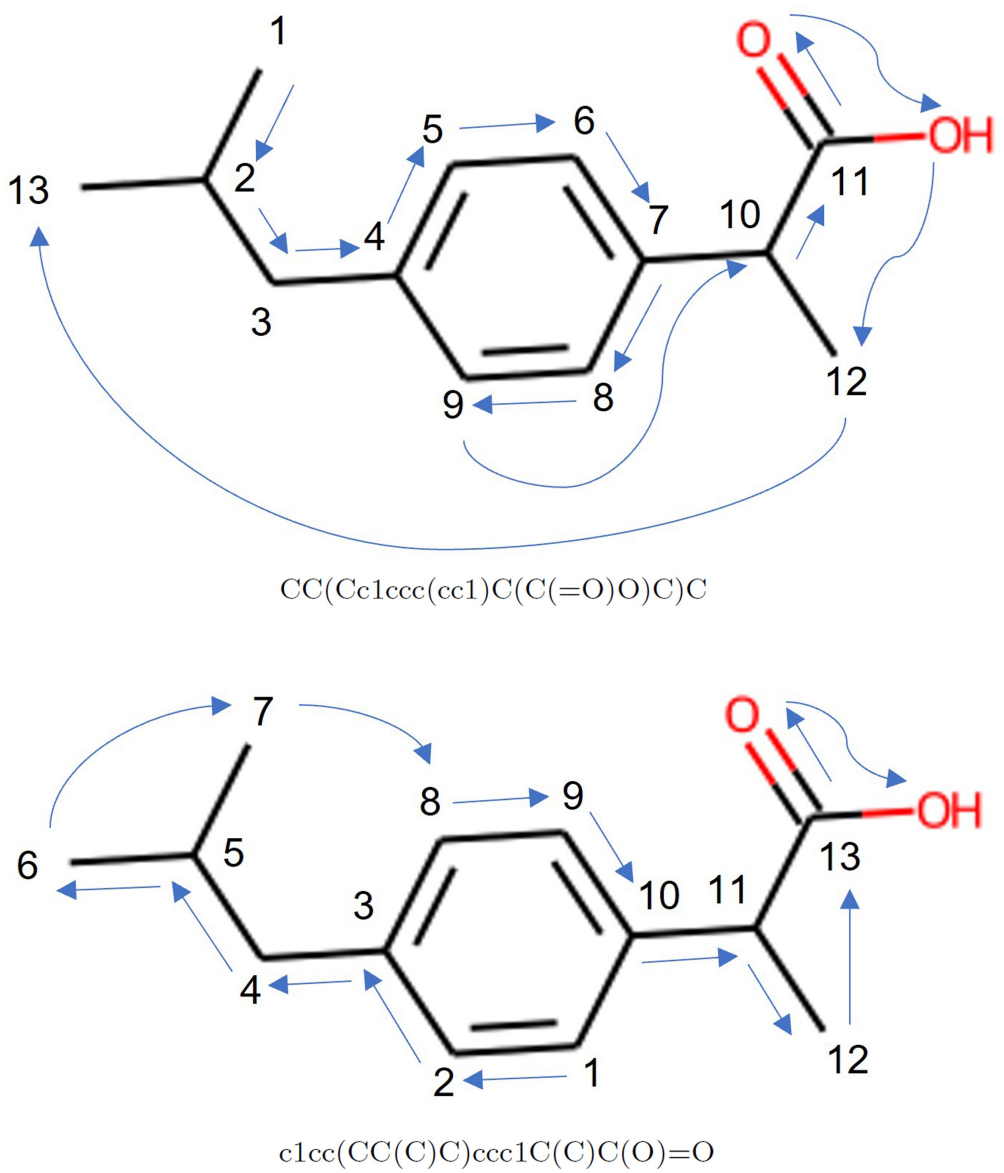
SMILES of ibuprofen with different atom numberings.

SimSon takes SMILES as its input and uses the SMILES enumeration technique proposed by (Bjerrum 2017) to augment SMILES data. The RDKit library (Landrum *et al.* 2013), an open-source cheminformatics software, is utilized for this SMILES enumeration process. To perform SMILES enumeration, the SMILES representation denoted as *x* of Molecule 1 is converted into a mol format, and the atom numbers within Molecule 1 are shuffled. This shuffling of atom numbers essentially changes the starting atom, as illustrated in the example shown in Fig. 3. Following the augmentation process, a new SMILES representation, *x*${}_{\mathrm{aug}}$, which represents Molecule 1, is generated from the mol format with different atom orderings. Both SMILES *x* and $x_{\mathrm{aug}}$ undergo encoding using a byte-pair encoding (BPE) tokenizer, a technique used in prior studies including Guo *et al.* (2021) and Chithrananda *et al.* (2020). This tokenizer converts the string representations into a numerical format, with a dictionary containing 300 tokens. The encoded vectors are then zero-padded to reach a maximum length of 512, and subsequently, they are embedded into $x,x_{\mathrm{aug}}\in R^{512\times 768}$.

### 3.3 Contrastive learning with transformer encoder

To capture meaningful molecular representations, the embedded SMILES sequences are passed through a Transformer encoder, where positional encodings are first added. SimSon uses a Transformer architecture with 2 layers and 8 attention heads to model dependencies between tokens in SMILES. The self-attention mechanism within the Transformer enables the extraction of molecular semantics by computing attention scores over all tokens in the sequence, explicitly establishing relationships between atoms and bonds. The details of the self-attention mechanism, including scaled dot-product attention and multi-head attention, are provided in the Supplementary Information.

Following the Transformer encoder, the processed outputs undergo global average pooling and pass through a linear layer to generate embedding vectors: *z* for SMILES *x* and $\hat{z}$ for its augmented version $x_{\mathrm{aug}}$. Both embeddings are processed by the same model and share identical weights. The objective of contrastive learning is to maximize the similarity between *z* and $\hat{z}$ while ensuring they remain distinct from embeddings of different molecules.

To achieve this, we use the normalized temperature-scaled cross entropy (NT-Xent) loss (Chen *et al.* 2020), which calculates cosine similarities to either minimize or maximize the distance between positive and negative pairs:


(1) $$L=-\text{log}\;\frac{\;\text{exp}(\text{sim}(z_{i},z_{\mathrm{aug}})/\tau )}{\sum_{k=1}^{2N}1[k\neq i]\;\text{exp}(\text{sim}\cdot (zi,z_{k})/\tau )}$$

where sim(·) represents cosine similarity:


(2) $$\text{sim}(A,B)=\frac{A\cdot B}{\left|A||B\right|}=\frac{\sum_{i=1}^{n}A_{i}B_{i}}{\sqrt{\sum_{i=1}^{n}{\left(A_{i}\right)}^{2}}\sqrt{\sum_{i=1}^{n}{\left(B_{i}\right)}^{2}}}$$

Here, *N* is the mini-batch size, and τ is the temperature parameter. The NT-Xent loss optimizes the contrastive learning process by enforcing positive pairs (*z*${}_{i}$, *z*${}_{\mathrm{aug}}$) to be similar while separating negative pairs ($z_{i}$, $z_{k}$) and ($z_{\mathrm{aug}}$, $z_{k}$), which correspond to different molecules. The individual contrastive loss terms are given by:


(3) $$L_{i}=-\text{log}\;\frac{\;\text{exp}(\text{sim}(z_{i},z_{\mathrm{aug}})/\tau )}{\sum_{k=1}^{N}1[k\neq i]\;\text{exp}(\text{sim}(zi,z_{k})/\tau )}$$

(4) $$L_{\mathrm{aug}}=-\text{log}\;\frac{\;\text{exp}(\text{sim}(z_{i},z_{\mathrm{aug}})/\tau )}{\sum_{k=1}^{N}1[k\neq \mathrm{aug}]\;\text{exp}(\text{sim}(\mathrm{zaug},z_{k})/\tau )}$$

Further details regarding the contrastive learning, negative pair selection, and loss function derivations can be found in the Supplementary Information.

### 3.4 Training details

SimSon is trained using a dataset comprising 1 million randomly selected unlabeled samples from PubChem. The pre-training of SimSon is conducted on a single 3090 GPU, spanning 16 epochs with a training time of 16 h. A batch size of 128 and a learning rate of 10${}^{-4}$ are used in the training process. The temperature parameter for the NT-Xent loss function is configured to be 0.2, and the optimizer used is AdamW.

## 4 Experiments

### 4.1 Experimental setup

The performance of SimSon is evaluated on eleven benchmark datasets sourced from MoleculeNet (Wu *et al.* 2018) to assess its effectiveness in molecular property prediction. These datasets include six classification tasks and five regression tasks, with detailed information available in the Supplementary Information. We primarily compare SimSon with other self-supervised learning-based models to evaluate its effectiveness in learning molecular representations. Additionally, we report the results of CMPNN (Song *et al.* 2020) and DMPNN (Yang *et al.* 2019), two supervised learning-based models, as well as KANO, which incorporates external knowledge graph data. The results of CMPNN, DMPNN, and KANO can be found in the Supplementary Information. Details regarding the experimental setup, including the data split method, classification approach, hyperparameters, and evaluation metrics, are also provided in the Supplementary Information.

### 4.2 Experimental results

Tables 1 and 2 present the classification and regression results across eleven benchmark datasets. SimSon achieves the best performance on seven out of eleven tasks, even though it uses a simple classification layer, whereas other models incorporate more complex classification architectures. This demonstrates that SimSon effectively learns SMILES representations for downstream tasks while maintaining strong generalization across the chemical space. This generalization ability is largely attributed to its training with randomized SMILES, which reduces overfitting by introducing controlled perturbations (Arús-Pous *et al.* 2019, 2020).

**Table 1.** Quantitative results of downstream classification tasks, with mean and standard deviation reported.^a^

Model	BBBP	Tox21	SIDER	ClinTox	HIV	BACE
ChemBERTa	88.04 (0.8590)	74.35 (4.8621)	60.02 (0.0157)	95.52 (1.4843)	69.46 (0.7709)	83.42 (0.0068)
GROVER-large	85.52 (3.9600)	74.26 (0.8603)		81.33 (9.5677)	70.17 (0.5801)	
GROVER-base	89.24 (1.2155)	70.94 (2.8930)		85.69 (0.4899)	72.62 (1.4382)	
MGSSL	68.36 (0.9850)	76.60 (0.3950)	56.17 (0.0100)	67.27 (1.2019)	75.89 (1.1579)	82.74 (0.0118)
MolCLR	81.83 (4.1910)	72.22 (3.3806)	58.23 (0.0951)	90.06 (1.8100)	**78.54 (1.3828)**	81.09 (0.0154)
GraphMVP	65.10 (0.0154)	**81.78 (0.0037)**	60.28 (0.0066)	80.78 (0.0208)	75.08 (0.0185)	69.62 (0.0625)
HiMol	86.21 (2.1866)	67.11 (0.7308)		59.32 (0.4636)	76.85 (0.9697)	
MolMVC	67.06 (0.0032)		56.81 (0.0126)	73.02 (0.1090)	67.51 (0.0076)	78.43 (0.0352)
MolLM	72.92 (0.0246)	79.34 (0.0020)		88.64 (0.0524)	69.05 (0.0518)	81.10 (0.0368)
SimSon (ours)	**92.30 (1.2124)**	76.99 (0.2166)	**63.93 (0.0227)**	**98.60 (0.7514)**	76.65 (0.6226)	**87.05 (0.0291)**

a AUC-ROC is used as the evaluation metric, and the best results are highlighted in bold (higher ↑ is better).

**Table 2.** Quantitative results of downstream regression tasks, with mean and standard deviation reported.^a^

Model	ESOL	FreeSolv	Lipophilicity	QM7	QM8
ChemBERTa	0.7688 (0.0753)	1.9428 (0.3818)	0.7577 (0.0039)	175.16 (1.1176)	0.0262 (0.0021)
GROVER-large	0.7966 (0.1715)	1.7937 (0.3923)	0.9148 (0.0784)		
GROVER-base	0.8463 (0.0265)	2.0610 (0.1486)	0.8691 (0.0629)		
MolCLR	0.7397 (0.0401)	1.5813 (0.0572)	**0.6350 (0.0111)**	84.33 (5.5607)	0.0208 (0.0012)
GraphMVP	0.7711 (0.0455)	**1.0821 (0.0532)**	0.6660 (0.0251)		
HiMol	0.7165 (0.0055)	1.3259 (0.0116)	0.6642 (0.0054)		
SimSon (ours)	**0.7052 (0.0322)**	1.5151 (0.0833)	0.8391 (0.0204)	**55.98 (0.143)**	**0.0120 (0.0010)**

a RMSE is used as the evaluation metric for ESOL, FreeSolv, and Lipophilicity, while MAE is used for QM7 and QM8. The best results are highlighted in bold (lower ↓ is better).

Although trained solely on SMILES data, SimSon performs competitively with graph-based methods, even without explicitly encoding molecular structures. Since SMILES inherently capture molecular connectivity and stereochemistry, self-attention mechanisms in SimSon enable it to model atom and bond relationships effectively. Furthermore, SimSon outperforms ChemBERTa, another Transformer-based model, due to its contrastive learning framework. Predictive pre-training methods such as ChemBERTa struggle to capture molecular-level semantics (Jaiswal *et al.* 2020), whereas SimSon’s contrastive approach, leveraging randomized SMILES, preserves global chemical information and enhances representation learning.

A comparison with contrastive learning-based methods such as MolCLR, MolMVC, and MolLM further highlights SimSon’s advantages. MolCLR primarily relies on local augmentations, such as masking or deleting atoms, bonds, and subgraphs, which can disrupt essential chemical semantics and weaken molecular representations. Augmenting molecular representations by dropping nodes or perturbing edges may violate chemical structures within molecules, as noted by (Fang *et al.* 2023), limiting the effectiveness of contrastive learning in MolCLR. MolMVC and MolLM extend contrastive learning to multiple molecular representations (1D, 2D, and 3D), but multi-view approaches may introduce inconsistencies when representations from different modalities are not perfectly aligned. In contrast, SimSon maintains representation consistency by leveraging randomized SMILES augmentations within the same molecular notation, ensuring that different views retain the same underlying semantics while introducing diversity.

SimSon achieves the best results in seven out of eleven datasets, indicating that while it does not consistently outperform all models, it remains a strong competitor in molecular property prediction. Its ability to perform at this level using only SMILES-based contrastive learning highlights the strength of learning molecular representations directly from sequence-based inputs. Unlike models that incorporate multi-modal learning strategies, SimSon relies solely on SMILES yet still delivers competitive results across multiple datasets. These findings reinforce the effectiveness of contrastive learning in capturing meaningful chemical representations without requiring additional structural modalities or external data.

## 5 Ablation studies

### 5.1 Number of parameters and pre-trained data

Detailed information regarding the number of model parameters and pre-training data can be found in the Supplementary Information. Transformer-based models, such as SimSon, ChemBERTa, GROVER, and MolLM, generally have a higher parameter count compared to GNN-based models. Additionally, most pre-trained models have been trained on datasets exceeding 1 million instances. To ensure a fair comparison, we pre-trained all models under similar conditions, minimizing the influence of dataset size and model parameters on performance. For GROVER and HiMol, we followed the pretraining steps as outlined in their respective GitHub repositories, using the same dataset for pre-training as was used for SimSon. After pre-training, the models were fine-tuned on task-specific datasets. In the case of ChemBERTa, we fine-tuned the model using the pre-trained weights from the model trained on 1 million data instances, as provided by (Chithrananda *et al.* 2020). For SimSon, we reduced the number of parameters by minimizing the embedding dimension, d_model, to the lowest feasible value.

As shown in Tables 3 and 4, SimSon achieves competitive results compared to other models trained on 1 million data instances, despite having the lowest number of parameters. This suggests that SimSon’s performance is not primarily determined by model size but rather by its ability to effectively learn SMILES representations through contrastive learning. This highlights the capability of SimSon to construct a meaningful SMILES embedding space, serving as a strong foundation for molecular property prediction.

**Table 3.** Quantitative results of classification tasks.^a^

Model	BBBP	Tox21	SIDER	ClinTox	HIV	BACE
ChemBERTa	89.44 (0.2836)	63.40 (7.3745)	0.5910 (0.0166)	97.84 (0.8041)	70.60 (1.2879)	78.85 (0.0124)
GROVER	89.57 (0.0289)	74.91 (0.1704)		91.63 (0.1001)	**78.23 (0.3676)**	
HiMol	87.17 (1.4652)	63.27 (2.4726)	**61.62 (0.0256)**	73.28 (2.8711)	77.64 (1.3054)	83.00 (0.0193)
SimSon-small	**89.97 (1.7416)**	**75.15 (0.9089)**	59.14 (0.0215)	**98.83 (0.1222)**	75.64 (1.4087)	87.07 (0.0498)

a Each model is pre-trained on 1 million data instances, and the number of parameters of SimSon is reduced. Mean and standard deviation are reported. AUC-ROC is used as the evaluation metric. The best results are highlighted in bold.

**Table 4.** Quantitative results of regression tasks.^a^

Model	ESOL	FreeSolv	Lipophilicity	QM7	QM8
ChemBERTa	0.8601 (0.0579)	2.0438 (0.0234)	1.014 (0.0285)	**61.76 (3.1425)**	0.0292 (0.0129)
GROVER	1.0057 (0.0006)	1.6967 (0.0001)	0.6747 (0.0003)		
HiMol	0.9358 (0.0096)	**1.3524 (0.0052)**	**0.6667 (0.0039)**	81.24 (8.1144)	0.0277 (0.0140)
SimSon-small	**0.8017 (0.0319)**	1.6120 (0.1449)	0.8918 (0.0261)	68.25 (3.2675)	**0.0174 (0.0007)**

a Each model is pre-trained on 1 million data instances, and the number of parameters of SimSon is reduced. RMSE is used as the evaluation metric for ESOL, FreeSolv, and Lipophilicity, while MAE is used for QM7 and QM8. The best results are highlighted in bold.

Despite its reduced number of parameters and pre-training data, SimSon-small consistently demonstrated competitive performance across most datasets. This result underscores the efficiency of its contrastive learning framework and its ability to learn effective molecular representations with fewer computational resources.

### 5.2 Effects of pre-training

Tables 5 and 6 compare the results of downstream tasks with and without pre-training. Significant improvements are observed in Tox21, SIDER, and BACE classification datasets, as well as in regression tasks. On average, there is an increase of 11.51% in classification tasks and a decrease of 23.6% in regression tasks when pre-trained with randomized SMILES. Notably, datasets like Tox21, which has 12 classes, and SIDER, which has 27 classes, show substantial improvement, suggesting that SimSon is particularly effective for datasets with limited data but a large number of classes.

**Table 5.** Comparison of classification results with and without pre-training.^a^

Model	BBBP	Tox21	SIDER	ClinTox	HIV	BACE
without pre-trained	88.61 (0.4493)	69.12 (0.6014)	53.77 (0.0090)	97.47 (0.4712)	74.96 (0.4990)	66.37 (0.0595)
pre-trained	92.30 (1.2124)	76.99 (0.2166)	63.93 (0.0227)	98.60 (0.7514)	76.65 (0.6226)	87.05 (0.0291)
simson-masked	89.06 (0.0278)	76.01 (0.0256)	55.45 (0.0085)	99.41 (0.0035)	72.51 (0.0256)	79.67 (0.0403)

a Mean and standard deviation are reported. AUC-ROC is used as the evaluation metric. Pre-trained results refer to contrastive learning between SMILES and randomized SMILES, whereas Pre-trained (masked) results refer to contrastive learning between SMILES representations with random masking.

**Table 6.** Comparison of regression results with and without pre-training.^a^

Model	ESOL	FreeSolv	Lipophilicity	QM7	QM8
without pre-trained	0.8717 (0.0211)	2.0044 (0.3023)	0.9482 (0.0339)	73.24 (8.964)	0.0198 (0.0013)
pre-trained	0.7052 (0.0322)	1.5151 (0.0833)	0.8391 (0.0204)	55.98 (0.143)	0.0120 (0.0010)
simson-masked	0.7071 (0.0771)	3.7035 (0.3744)	0.8261 (0.0295)	67.38 (0.8259)	0.0174 (0.0009)

a Mean and standard deviation are reported. RMSE is used for ESOL, FreeSolv, and Lipophilicity datasets, while MAE is used for QM7 and QM8 datasets. Pre-trained results refer to contrastive learning between SMILES and randomized SMILES, whereas Pre-trained (masked) results refer to contrastive learning between SMILES representations with random masking.

To assess whether the improvements in SimSon stem from the proposed contrastive learning with randomized SMILES, contrastive learning itself using masked SMILES, or the model architecture, an additional pre-training strategy was explored. Specifically, SimSon was pre-trained using a masking augmentation strategy where up to 25% of the SMILES string was randomly masked for contrastive learning. As shown in Tables 5 and 6, pre-training with randomized SMILES outperforms masked SMILES augmentation in most datasets, highlighting the effectiveness of leveraging randomized SMILES as an augmentation method. A key reason for this difference is that masked SMILES augmentation relies on local perturbations, such as the deletion of atoms or bonds, which may disrupt molecular semantics (Fang *et al.* 2023). In contrast, randomized SMILES preserves global molecular semantics while introducing controlled perturbations, making it a more effective strategy for contrastive learning.

Pre-training with masked SMILES achieves the best results in Lipophilicity and ClinTox, suggesting that localized perturbations can be more effective than preserving overall molecular structures for certain properties. Lipophilicity, influenced by atomic interactions, and ClinTox, which focuses on toxicity, may benefit from learning atom- and bond-level features rather than global molecular structure.

In some cases, pre-training has minimal impact, as seen in the HIV, ClinTox, and QM8 datasets, where models without pre-training still perform competitively. This suggests that direct supervised learning may suffice for tasks with diverse or well-defined patterns, reducing the necessity for additional contrastive learning.

Overall, these results highlight the benefits of pre-training with randomized SMILES, particularly for datasets with a large number of classes. While masked augmentation offers advantages in specific cases, leveraging randomized SMILES allows SimSon to capture a more comprehensive understanding of molecular structures, leading to stronger generalization across diverse molecular property prediction tasks.

### 5.3 Similarities of embedding vectors

Both SimSon and ChemBERTa are Transformer-based models designed to learn SMILES representations, but they differ in their training methods. ChemBERTa uses a predictive self-supervised learning approach, while SimSon utilizes contrastive learning with randomized SMILES. SMILES is not always in its canonical form in real-world data, so handling variations in SMILES representations is crucial for ensuring model robustness and generalization across different molecular representations. To investigate this, cosine similarity between embedding vectors for SMILES and their randomized versions was computed.

To assess the consistency of the models when non-canonical SMILES are input, each downstream dataset was fine-tuned with its corresponding canonical SMILES data. The fine-tuned models then output embedding vectors when SMILES and randomized SMILES are input. The cosine similarity between these embedding vectors was calculated using the formula defined in Equation (3). As shown in Tables 7 and 8, SimSon exhibits higher cosine similarity scores compared to ChemBERTa, with an average similarity of 0.8482, while ChemBERTa’s average is 0.7590. This difference is likely due to ChemBERTa’s reliance on a masked language model (MLM) loss function, which introduces a bias toward learning a representation specific to canonical SMILES (Carlsson *et al.* 2021). This bias can hinder the model’s ability to generalize across different SMILES variations, as ChemBERTa struggles with representations that deviate from the canonical form.

**Table 7.** Cosine similarities between embedding vectors of SMILES and randomized SMILES for classification tasks.^a^

Model	BBBP	Tox21	SIDER	ClinTox	HIV	BACE
ChemBERTa	0.5695 (0.2533)	0.9208 (0.0206)	0.7158 (0.0588)	0.8327 (0.2856)	0.8921 (0.0453)	0.4202 (0.0158)
SimSon	0.9334 (0.1479)	0.9813 (0.0661)	0.8215 (0.0759)	0.9133 (0.2382)	0.9330 (0.0203)	0.5088 (0.0320)

a ChemBERTa and SimSon are compared to assess the robustness of their learned SMILES representations and syntax. Mean and standard deviation are reported.

**Table 8.** Cosine similarities between embedding vectors of SMILES and randomized SMILES for regression tasks.^a^

Model	ESOL	FreeSolv	Lipophilicity	QM7	QM8
ChemBERTa	0.8739 (0.0491)	0.8387 (0.0961)	0.7785 (0.0768)	0.8198 (0.0202)	0.6869 (0.0382)
SimSon	0.9876 (0.0361)	0.8527 (0.1095)	0.8333 (0.1369)	0.8215 (0.0759)	0.7440 (0.0456)

a The performance of ChemBERTa and SimSon is evaluated to determine their stability in learning SMILES representations and syntax. Mean and standard deviation are reported.

SimSon, on the other hand, is pre-trained using randomized SMILES, which enables it to generalize across diverse molecular representations. Even though SimSon is fine-tuned with canonical SMILES data in downstream tasks, its contrastive learning framework allows the model to retain stability when exposed to variations in SMILES representations. This helps reduce task-specific bias and ensures more consistent embedding vectors. As a result, SimSon outputs similar embedding vectors even when non-canonical SMILES are input, demonstrating its learned stability in SMILES syntax. This contributes to SimSon’s higher cosine similarity scores compared to ChemBERTa.

Overall, the results demonstrate the robustness of SimSon in handling SMILES variability. The pre-training using contrastive learning with randomized SMILES mitigates inconsistencies introduced by different SMILES generation algorithms, enhancing its ability to generalize across diverse molecular representations.

## 6 Conclusion

In this study, we introduced a self-supervised framework that combines contrastive learning with the SMILES enumeration technique to learn the SMILES representation effectively. SimSon demonstrates its capability to generalize the chemical space and exhibits robustness in molecular property predictions, as demonstrated through our experiments. We demonstrate that contrastive learning with randomized SMILES and a self-attention mechanism can capture enhanced molecular semantics. This approach extracts inherent molecular structural information from SMILES, allowing SimSon to achieve competitive results compared to graph-based methods without the need for implicit structural encoding. Moreover, SimSon offers the advantage of efficient memory usage during training as it operates solely on SMILES data. Furthermore, it excels in leveraging the wealth of available SMILES data, which is widely accessible in molecular databases, unlike other molecular representations such as IUPAC.

In future work, we plan to scale up SimSon by training it on a larger dataset containing 10 million samples to further enhance its generalization capabilities, given that Transformer-based models tend to perform better with more extensive training data due to their inductive bias. We also intend to explore how SimSon can be applied to tasks such as drug–drug interaction prediction and drug design, expanding its potential applications in the field of bioinformatics and chemoinformatics.

## Supplementary Material

btaf275_Supplementary_Data
