# Supplementary material for: SimSon: simple contrastive learning of SMILES for molecular property prediction
Source: Bioinformatics. 2025 May 8;41(5):btaf275. doi: 10.1093/bioinformatics/btaf275 (PMC12124188; doi:10.1093/bioinformatics/btaf275)
Supplement: btaf275_Supplementary_Data [file btaf275_supplementary_data.pdf]

Supplementary Information for: SimSon: Simple Contrastive Learning of SMILES for Molecular Property Prediction

Chae Eun Lee[1], Jin Sob Kim[2], Jin Hong Min[3], Sung Won Han[*]

[1, 2, 3, *] Department of Industrial and Management Engineering, Korea University

[*]Corresponding author: Sung Won Han

1. Self-Attention Mechanism in Transformer

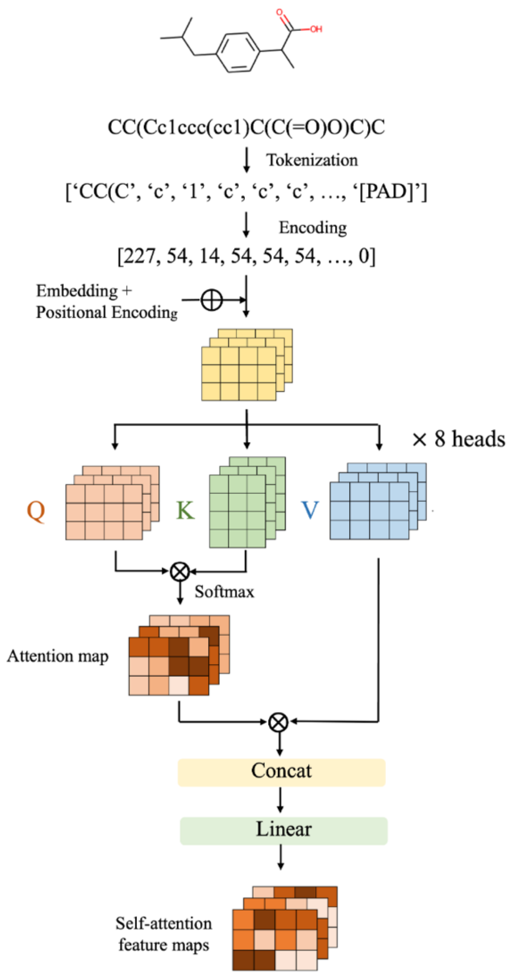

Fig. 1: The detailed illustration of the self-attention mechanism in Transformer. Q, K, and V correspond to the query, key, and value vectors, respectively. Q, K, and V are trained with backpropagation and are used to compute attention scores. Each Q, K, and V vector has 8 vectors as the number of heads is set to 8 in Simson

The Transformer encoder in SimSon employs self-attention to capture dependencies between tokens in SMILES sequences, allowing the model to establish explicit relationships between atoms and bonds. The self-attention mechanism consists of scaled dot-product attention and multi-head attention, which together enhance the model's ability to extract meaningful molecular representations.

Self-attention operates by computing attention scores for each token relative to all other tokens in a sequence. Given input embeddings x, the model first projects them into three distinct representations: query Q, key K, and value V, using learned linear transformations. The dimensions of Q and K are denoted as d_k, while V has a dimension of d_v. The attention scores are computed using scaled dot-product attention as follows:

$$Attention(Q,K,V) = softmax\left(\frac{QK^T}{\sqrt{d_k}}\right)V$$

Here, the dot product between Q and K^T measures the similarity between query and key vectors, and the division by $\sqrt{d_k}$ stabilizes the gradients during training. Applying the softmax function normalizes the attention scores, ensuring that the model selectively attends to relevant tokens in the sequence. The weighted sum of the values V is then computed, generating refined representations that integrate contextual information from other tokens.

Instead of relying on a single set of attention weights, multi-head attention enables the model to capture diverse interaction patterns by applying multiple independent attention operations in parallel. The input embeddings are linearly projected into multiple sets of queries, keys, and values, performing attention computation h times:

$$MultiHead(Q,K,V) = Concat(head_1, head_2, \dots, head_h)W^O$$

where each attention head computes scaled dot-product attention independently. The outputs from all heads are then concatenated and passed through a final linear transformation W^O. This approach allows the model to capture different aspects of molecular structure simultaneously, improving representation learning.

SimSon utilizes 8 attention heads, with the dimensions of Q, K, V set to $\mathbb{R}^{512X96}$, meaning that each attention head processes a 96-dimensional subspace of the 768-dimensional input embeddings. After attention computation, the outputs from all heads are concatenated, yielding final representations of size $\mathbb{R}^{512X768}$, which are subsequently passed through a linear transformation.

The self-attention mechanism within SimSon's Transformer encoder enables efficient learning of molecular semantics by assigning varying importance to different tokens, thereby capturing both local and long-range dependencies in SMILES representations. This enhances the model's ability to generate stable molecular embeddings, even when presented with different SMILES notations for the same molecule.

2. Contrastive Learning

The embeddings z and $\hat{z}$ obtained from the linear layer represent high-dimensional vectorized representations of molecular structures. Specifically, z corresponds to the embedding of the original SMILES sequence, while $\hat{z}$ corresponds to the embedding of its augmented version, which is generated using the SMILES enumeration technique. Since both embeddings are derived from the same molecular structure, they are expected to have high similarity scores.

The contrastive learning process involves constructing positive and negative pairs. Positive pairs consist of an original SMILES sequence and its augmented counterpart, encouraging the model to learn invariant molecular representations. In contrast, negative pairs are formed by pairing SMILES sequences from different molecules within the same mini-batch, ensuring that distinct molecular structures remain distinguishable. The model generates negative pairs for both embeddings, leading to multiple negative pairs within a mini-batch.

The NT-Xent loss function is computed based on cosine similarity and is designed to push positive pairs closer together while increasing the distance between negative pairs. The contrastive loss is computed separately for the original SMILES embedding and its augmented counterpart, ensuring consistency in learned representations. Indicator functions are used to exclude positive pairs from the denominator of the loss function. The temperature parameter τ controls the sharpness of the similarity distribution, where lower values increase contrast between positive and negative pairs, while higher values lead to a smoother similarity distribution.

This contrastive learning framework enables the model to learn chemically meaningful representations of molecules directly from SMILES sequences without requiring additional structural transformations.

3. Experimental Setup

We split each dataset into training, validation, and testing sets in an 80/10/10 ratio, using either scaffold or random splits, as recommended by MoleculeNet. For downstream evaluation, a classification head consisting of a single linear layer was appended to the model, and fine-tuning was conducted. The hyperparameters used for fine-tuning are summarized in TABLE 1, where optimal values were determined via grid search for each dataset.

The model was evaluated on eleven benchmark datasets, comprising six classification and five regression tasks. Details of these datasets can be found in TABLE 2. To ensure a robust comparison, results were averaged across three different random seeds. Our evaluation includes five self-supervised pre-trained models, and an overview of these baseline models is provided in TABLE 3. To maintain fairness, we reproduced the downstream results of each model using their publicly available pre-trained weights from GitHub. The fine-tuning procedures and hyperparameters closely follow those originally employed for each model, except for ChemBERTa, where a single classification layer was attached for fine-tuning. Additionally, all models were trained and evaluated using the same dataset splits to ensure consistency.

SimSon was trained for a maximum of 500 epochs; however, it was not necessarily trained for the full 500 epochs, as early stopping was applied based on validation performance to prevent overfitting.

Results were excluded if a benchmark dataset did not have an officially provided generation script or a preprocessed version compatible with the model's input format, ensuring that only datasets with available standardized processing were evaluated.

Table 1. Hyperparameters used to fine-tune downstream tasks.

Hyper-parameters	Description	Range
batch_size	Batch size used to train the model	{16, 32, 64, 128}
lr	Initial learning rate	{1e-6, 1e-4}
dropout	Dropout ratio in the classification layer	{0, 0.1, 0.2}
d_model	Dimensionality of the input	{200, 768}
warm_epoch	Number of epochs to restart the learning rate	{0, 1, 2, 3, 4, 5}

Table 2. Detailed information on benchmark datasets.

Task Type	Dataset	Description	# Tasks	# Compound	Split Method	Metric
Classification	BBBP	Blood-brain barrier penetration	1	2,039	Scaffold	AUC-ROC
	Tox21	Toxicity measurements on biological targets	12	7,831	Random	AUC-ROC
	SIDER	Marketed drugs and their adverse drug reactions	27	1,427	Random	AUC-ROC
	ClinTox	Clinical trials for toxicity	2	1,484	Scaffold	AUC-ROC
	HIV	Abilities to inhibit HIV replication	1	41,127	Scaffold	AUC-ROC
	BACE	Binding affinity data for BACE-1 inhibitors	1	1,513	Scaffold	AUC-ROC
Regression	ESOL	Water solubility	1	1,128	Random	RMSE
	FreeSolv	Hydration free energy of small molecules in water	1	642	Random	RMSE
	Lipophilicity	Octanol/water distribution coefficient	1	4,200	Random	RMSE
	QM7	Electronic properties of small molecules from DFT calculations	1	7,160	Random	MAE
	QM8	Electronic spectra and excited state energies of molecules	12	21,786	Random	MAE

Table 3. Summary of self-supervised baseline models.

Model	Representation	Self-supervised method	Number of parameters	Number of pre-trained data
ChemBERTa (1)	SMILES	Predictive	3,427,440	77M
GROVER-large (2)	Graph	Predictive	107,705,236	11M
GROVER-base (2)	Graph	Predictive	48,790,436	11M
MGSSL (3)	Graph	Predictive	48,790,436	250K
MolCLR (4)	Graph	Contrastive	1,858,201	10M
GraphMVP (5)	Graph	Contrastive	2,207,846	50K
HiMol (6)	Graph	Predictive	1,858,201	250K
KANO (7)	Graph	Contrastive	5,480,449	250K
MolMVC (8)	SMILES, Graph	Contrastive	2,178,806	3M
MolLM (9)	SMILES, Graph	Contrastive	7,436,843	160K
SimSon	SMILES	Contrastive	50,003,852	1M
SimSon-small	SMILES	Contrastive	13,606,700	1M

4. Evaluation Metrics

We utilized three key evaluation metrics: area under the receiver operating characteristic curve (AUC-ROC), root-mean-squared error (RMSE), and mean absolute error (MAE) to compare model performance across classification and regression tasks.

For classification tasks, AUC-ROC was used to assess the model's binary classification performance. AUC-ROC serves as a metric to assess the binary classification performance of the model and to determine the optimal cut-off value for classifying positive and negative classes (10). This metric quantifies the area under the ROC curve, where the ROC curve represents the plot of sensitivity against (1 - specificity) of a diagnostic test. Sensitivity signifies the proportion of subjects with the target condition who test positive, while specificity represents the proportion of subjects who test negative despite not having the target condition. Thus, a high AUC indicates that the model performs well in classification tasks, with an AUC exceeding 0.8 typically considered acceptable. AUC-ROC proves to be a more robust metric compared to accuracy, especially for classification problems characterized by imbalanced class distributions because accuracy can be misleading in such scenarios as it is highly dependent on the proportion of positive and negative classes even though classification problems with equivalent positive and negative classes are rare. Because the accuracy may be high even if the sensitivity and specificity are low, the AUC-ROC is used as a metric to compare the classification results.

For regression tasks, we employ RMSE as the evaluation metric, defined as (11):

$$RMSE = \sqrt{\frac{1}{n}\sum_{i=1}^{n}(y_i - \hat{y}_i)^2}$$

where y_i is the true value and $\hat{y}_i$ is the model prediction. RMSE measures the average magnitude of error, giving higher weight to larger errors, making it particularly sensitive to outliers. A lower RMSE indicates more accurate predictions.

MAE is an alternative regression metric defined as:

$$MAE = \frac{1}{n}\sum_{i=1}^{n}|y_i - \hat{y}_i|$$

Unlike RMSE, MAE assigns equal weight to all errors, providing a more interpretable measure of absolute prediction error.

5. Experimental Results of DMPNN, CMPNN and KANO

Table 4. Quantitative results of downstream classification tasks, with mean and standard deviation reported. AUC-ROC is used as the evaluation metric

Model	BBBP	Tox21	SIDER	ClinTox	HIV	BACE
DMPNN	73.12 (0.0157)	78.50 (0.0035)	0.5890 (0.0237)	94.96 (0.0011)	73.16 (0.0296)	78.30 (0.0545)
CMPNN	95.04 (0.0131)	84.27 (0.0057)	62.32 (0.0432)	90.78 (0.0043)	0.8265 (0.0124)	0.8424 (0.0116)
KANO	95.94 (0.0052)	85.08 (0.0094)	65.73 (0.0045)	88.52 (0.0436)	80.23 (0.0123)	85.63 (0.0072)

Table 5. Quantitative results of downstream regression tasks, with mean and standard deviation reported. RMSE is used for ESOL, FreeSolv, and Lipophilicity datasets, while MAE is used for QM7 and QM8 datasets.

Model	ESOL	FreeSolv	Lipophilicity	QM7	QM8
DMPNN	0.7641 (0.0933)	0.9636 (0.1319)	0.5796 (0.0105)	86.50 (1.5487)	0.0322 (1.5E-5)
CMPNN	0.7222 (0.0340)	1.4274 (0.4440)	0.8148 (0.3428)	57.63 (2.2579)	0.0134 (1.4E-4)
KANO	0.5603 (0.0199)	1.0013 (0.1034)	0.5818 (0.0341)	101.51 (2.2791)	0.0004 (0.0004)

To provide a more comprehensive analysis, we report the results of DMPNN (12) and CMPNN (13), which are supervised learning models, alongside KANO, which incorporates external knowledge. KANO consistently achieves strong results, outperforming many self-supervised models. DMPNN and CMPNN, despite being supervised models, show competitive results compared to self-supervised models, excelling on the BBBP, Tox21, and Lipophilicity datasets. This result suggests that when labeled data is sufficiently available for fine-tuning, supervised models can learn more task-specific features compared to pre-trained models, which rely on general-purpose representations. While self-supervised learning enables models to generalize across diverse molecular properties, supervised training directly optimizes for the target task, leading to superior performance in specific scenarios where high-quality labels are abundant. Nevertheless, pre-training remains essential for scenarios where labeled data is scarce or highly heterogeneous, providing a strong foundation for downstream adaptation.

6. Visualization of Embedding Space

We visualized the embedding spaces of SimSon, ChemBERTa, MolCLR, HiMol, and MGSSL for the BBBP and ESOL datasets using t-SNE (14) to reduce the dimensionality to two dimensions. As shown in Fig. 2 and Fig. 3, SimSon exhibits well-clustered data points with similar classes, indicating that molecules with similar properties are well aligned in the chemical space. Although ChemBERTa and HiMol also demonstrate good clustering of BBBP data points within the same classes, SimSon showcases denser clusters than other models in the ESOL dataset. These dense clusters in the BBBP and ESOL datasets suggest that SimSon excels in capturing molecular representations.

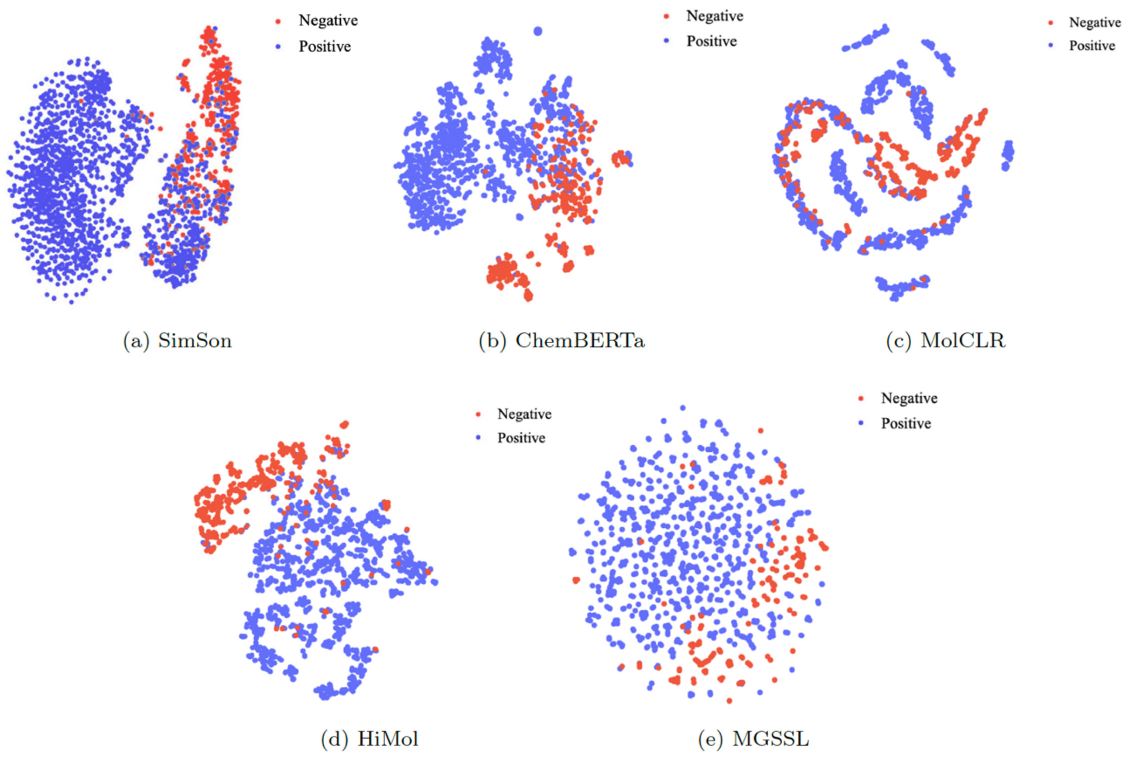

Fig. 2: Visualization of the embedding space of BBBP dataset. Red and blue colors represent positive and negative to blood-brain barrier penetration, respectively.

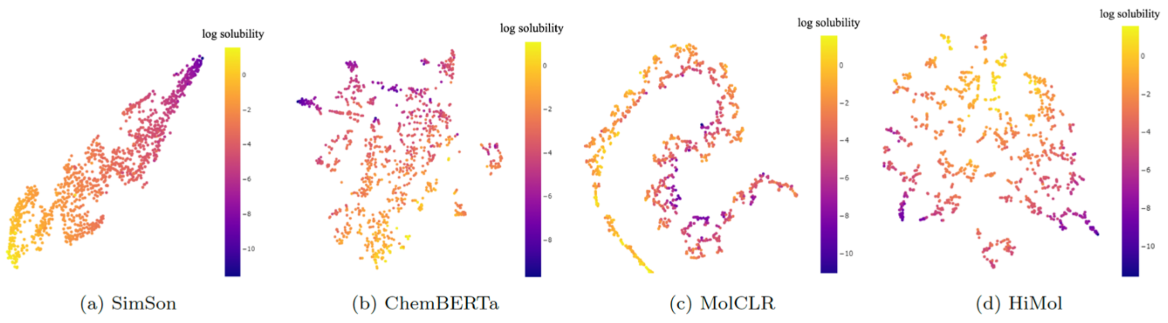

Fig. 3: Visualization of the embedding space of ESOL dataset. The color bar represents water solubility (log solubility in moles per liter).

References

1. Chithrananda, S., Grand, G. & Ramsundar, B. Chemberta: Large-scale self-supervised pretraining for molecular property prediction. ArXiv Preprint ArXiv:2010.09885. (2020)

2. Rong, Y., Bian, Y., Xu, T., Xie, W., Wei, Y., Huang, W. & Huang, J. Self-supervised graph transformer on large-scale molecular data. Advances In Neural Information Processing Systems. 33 pp. 12559-12571 (2020)

3. Zhang, Z., Liu, Q., Wang, H., Lu, C. & Lee, C. Motif-based graph self-supervised learning for molecular property prediction. Advances In Neural Information Processing Systems. 34 pp. 15870-15882 (2021)

4. Wang, Y., Wang, J., Cao, Z. & Barati Farimani, A. Molecular contrastive learning of representations via graph neural networks. Nature Machine Intelligence. 4, 279-287 (2022)

5. Liu, S., Wang, H., Liu, W., Lasenby, J., Guo, H. & Tang, J. Pre-training molecular graph representation with 3d geometry. ArXiv Preprint ArXiv:2110.07728. (2021)

6. Zang, X., Zhao, X. & Tang, B. Hierarchical molecular graph self-supervised learning for property prediction. Communications Chemistry. 6, 34 (2023)

7. Fang, Y., Zhang, Q., Zhang, N., Chen, Z., Zhuang, X., Shao, X., Fan, X. & Chen, H. Knowledge graph-enhanced molecular contrastive learning with functional prompt. Nature Machine Intelligence. 5, 542-553 (2023)

8. Huang, Z., Fan, Z., Shen, S., Wu, M. & Deng, L. MolMVC: Enhancing molecular representations for drug-related tasks through multi-view contrastive learning. Bioinformatics. 40, ii190-ii197 (2024)

9. Tang, X., Tran, A., Tan, J. & Gerstein, M. MolLM: a unified language model for integrating biomedical text with 2D and 3D molecular representations. Bioinformatics. 40, i357-i368 (2024)

10. Nahm, F. Receiver operating characteristic curve: overview and practical use for clinicians. Korean Journal Of Anesthesiology. 75, 25-36 (2022)

11. Hodson, T. Root-mean-square error (RMSE) or mean absolute error (MAE): When to use them or not. Geoscientific Model Development. 15, 5481-5487 (2022)

12. *Yang, K., Swanson, K., Jin, W., Coley, C., Eiden, P., Gao, H., Guzman-Perez, A., Hopper, T., Kelley, B., Mathea, M. & Others Analyzing learned molecular representations for property prediction. Journal Of Chemical Information And Modeling. 59, 3370-3388 (2019)*

13. Song, Y., Zheng, S., Niu, Z., Fu, Z., Lu, Y. & Yang, Y. Communicative Representation Learning on Attributed Molecular Graphs.. IJCAI. 2020 pp. 2831-2838 (2020)

14. Maaten, L. & Hinton, G. Visualizing data using t-SNE. Journal Of Machine Learning Research. 9 (2008)
